# Application of Size and Maturation Functions to Population Pharmacokinetic Modeling of Pediatric Patients

**DOI:** 10.3390/pharmaceutics11060259

**Published:** 2019-06-03

**Authors:** Hyun-moon Back, Jong Bong Lee, Nayoung Han, Sungwoo Goo, Eben Jung, Junyeong Kim, Byungjeong Song, Sook Hee An, Jung Tae Kim, Sandy Jeong Rhie, Yoon Sun Ree, Jung-woo Chae, JaeWoo Kim, Hwi-yeol Yun

**Affiliations:** 1 Department of Pharmaceutics, Ernest Mario School of Pharmacy, Rutgers, The State University of New Jersey, Piscataway, NJ 08854, USA; hyunmoon.back@rutgers.edu (H.-m.B.); jongbong.lee@rutgers.edu (J.B.L.); 2 College of Pharmacy, Seoul National University, Gwanak-ro 1, Gwanakgu, Seoul 08826, Korea; hans1217@snu.ac.kr; 3 College of Pharmacy, Chungnam National University, Daehak-ro 99, Yuseonggu, Daejeon 34134, Korea; swgoo@cnu.ac.kr (S.G.); jyeongkim@cnu.ac.kr (J.K.); jwchae@cnu.ac.kr (J.-w.C.); 4 Ministry of Food and Drug Safety, Osongsangmyung 2-ro 187, Cheongju, Chungbuk 28159, Korea; ebjung@korea.kr; 5 JW Pharmaceutical Corp., Drug Discovery Center, Nambusunhwan-ro 2477, Seochogu, Seoul 06725, Korea; bjsong@jw-pharma.co.kr; 6 College of Pharmacy, Wonkwang University, Iksandae-ro 460, Iksan, Jeonbuk 54538, Korea; shan7@wku.ac.kr; 7 Department of Pharmacy, Kyunghee University Hospital at Gang-dong, Dongnam-ro 892, Kangdonggu, Seoul 05278, Korea; jtkim@khnmc.or.kr; 8 College of Pharmacy, Ewha Womans University, Ewhayeodae-gil 52, Seoul 03760, Korea; sandy.rhie@ewha.ac.kr; 9 Department of Pharmacy, Yonsei University Health System, Yonsei-ro 50-1, Seodaemun-gu, Seoul 03722, Korea; YOONSUN@yuhs.ac; 10 Yangji Hospital, 1636 Nambusunhwan-ro, Gwanak-gu, Seoul 08779, Korea

**Keywords:** size function, maturation function, pharmacometrics, pediatrics, cyclosporin, phenobarbital, vancomycin

## Abstract

Traditionally, dosage for pediatric patients has been optimized using simple weight-scaled methods, but these methods do not always meet the requirements of children. To overcome this discrepancy, population pharmacokinetic (PK) modeling of size and maturation functions has been proposed. The main objective of the present study was to evaluate a new modeling method for pediatric patients using clinical data from three different clinical studies. To develop the PK models, a nonlinear mixed effect modeling method was employed, and to explore PK differences in pediatric patients, size with allometric and maturation with Michaelis–Menten type functions were evaluated. Goodness of fit plots, visual predictive check and bootstrap were used for model evaluation. Single application of size scaling to PK parameters was statistically significant for the over one year old group. On the other hand, simultaneous use of size and maturation functions was statistically significant for infants younger than one year old. In conclusion, population PK modeling for pediatric patients was successfully performed using clinical data. Size and maturation functions were applied according to established criteria, and single use of size function was applicable for over one year ages, while size and maturation functions were more effective for PK analysis of neonates and infants.

## 1. Introduction

In medicine, the general concept that “adults are big children” and “children are old babies” [1] can result in physiological differences between babies and adults being ignored when researchers handle large pediatric datasets. Although this concept is partially consistent with structural aspects of the body, pediatric patients, especially neonates, undergo rapid changes in their organs during maturation [2], which can bring big differences to physiological conditions.

In general, children and adults display differences in pharmacokinetics (PKs) including absorption, distribution, metabolism and excretion (ADME) of endogenous and exogenous substances [3,4,5,6]. Young patients can absorb nutrients and drugs differently due to active assimilation, and distribution can also differ from that in adults and can vary with age due to differences in the amount of plasma proteins, and the relative amounts of fluid, fat and tissues [7]. Furthermore, it can be difficult to predict metabolism in young people, regardless of the type of xenobiotic, because enzyme activity is closely related to enzyme maturity. Important metabolic pathways such as the cytochrome P450 system in neonates and infants are not as efficient in children, especially during gestation and between six and twelve months of age [6,8,9]. Therefore, the metabolic ability to process xenobiotics can alter with organ maturity, and secretion ability can also be decreased in the early postnatal period [6,9,10].

Due to the above differences in PK characteristics in pediatric patients, dosage is primarily selected based on labeling. However, appropriate doses have not been determined for many drugs due to difficulties in performing clinical trials on pediatric patients. When appropriate dosage information is unavailable, many clinicians use Young or Clark equations to calculate dose based on age, body weight, body surface area (BSA) and other parameters [11]. However, such equations tend to underestimate the importance of body mass, which can often result in inappropriate dosage for children [12,13].

To overcome this limitation, an alternative method can be used to calculate the pediatric dose using PK parameters and compartmental modeling. PK parameters such as volume of distribution (*V_d_*) and clearance (*CL*) are related to physiological differences that arise due to age and maturation [14,15]. In clinical situations, *V_d_* and *CL* can be crucial factors for calculating appropriate initial and maintenance dosage, respectively, and plasma concentration curves are also largely dependent on these two parameters [16]. Conceptually, both of these PK parameters may be related to physiological conditions such as body size, maturation of body function and organ function, but few previous studies have quantified the relationships between PK parameters and such physiological conditions [17]. According to previous reports, body size is an important predictor of *CL* and *V_d_* in pediatric patients, and the fractal concept is a key factor in determination of accurate *CL* and *V_d_* values to allow quantification of the relationship between the mass/structure of an organ and size (*F_size_*) [18,19,20,21]. The *F_size_* parameter can be expressed using Equation (1):

(1) $$F_{size}=a\cdot {\left(Body\;mass\right)}^{Power}$$

where *a* and *Power* are the allometric coefficient and exponent, respectively. 

Other factors affecting PKs in pediatrics include the maturation of organs. As previously reported [22], there exists a nonlinear relationship between organ maturity and post-conceptual age (PCA), which can be explained using a sigmoidal maximum response (*E_max_*) model of early slow growth and subsequent faster growth according to Equation (2):

(2) $$F_{mat}=\frac{PCA^{Hill}}{PCA^{Hill}+TM_{50}{}^{Hill}}$$

where *F_mat_* is the maturation function value, which is the ratio of pediatric PK parameters to adult PK parameters, *PCA* is post-conception age, *TM*_50_ is the PCA when reaching 50% of adult PK parameters and *Hill* is the coefficient associated with the slope of the maturation profile [22]. 

Despite concerns about differences in body size and maturity between adults and pediatrics, weight-based linear extrapolation or recommended dose for specific age groups is often used due to easy applicability. However, some previous studies report that adverse drug reactions (ADRs) are related to inappropriate dosage for pediatric patients, especially for drugs with narrow therapeutic ranges, leading to ineffective treatment and even fatality in some cases [23,24]. Recently, population PK analysis has been applied in an attempt to overcome these problems, and a new method involving size function with allometric scaling and maturation functions based on the Michaelis–Menten type was established for customized dose setting for pediatric patients [2,25,26,27,28,29]. Therefore, the main objectives of this study were to evaluate this new method in pediatric patients following administration of three narrow therapeutic range drugs, cyclosporine A (CsA), phenobarbital (PHB) and vancomycin (VAN), and determine the optimal pediatric dose of these drugs based on size and maturity. 

## 2. Methods

### 2.1. Categorization of Pediatric Patients Based on Physiological Conditions

To categorize pediatrics, we separated them into groups based on physiological conditions. As previously reported, patients could be stratified into five categories: preterm neonates, term neonates, infants, children and adolescents, equating to 37 weeks of gestation, 0 to 4 weeks, 1 month to 1 year, 1 to 12 years and 12 to 16 or 18 years, respectively [7]. These five categories were used throughout the current study.

### 2.2. Data Collection for CsA, PHB and VAN

Analysis of CsA was performed to evaluate its efficacy and safety in patients from infants to children with retinoblastoma in Yonsei University Hospital, as a retrospective study that was approved by the institutional review board of Yonsei University Health System (IRB file no. 4-2015-0372, 21.06.2015). In this case, CsA was used to enhance efficacy of chemotherapy of retinoblastoma via its p-glycoprotein (P-gp) inhibitory effect [30,31]. A high dose of CsA was infused over 24 h and started 3 h before the first dose of chemotherapy on day 1 and 2 [32]. Blood samples for CsA analysis were collected at 20 h after administration of CsA on days 1 and 2. 

Analysis of PHB was performed to monitor seizure control in patient from preterm neonate to infants. This study was proceeded in the neonatal intensive care unit (NICU) of Kyunghee University at Gang-dong, and was approved by the institutional review board in Kyunghee University Hospital (IRB file no. 2015-01-026-002, 29.04.2016). To control seizures in pediatric patients, PHB was used under the general guidelines [33] at an initial dose of 15–20 mg/kg followed by a maintenance dose of 3–5 mg/kg/day after 12–24 h the initial dose. Blood samples were taken between 5 min and 3 h before the subsequent dose.

Analysis of VAN was performed as a retrospective study to evaluate its use in patient from preterm neonates to infants and was approved by Chungnam National University Hospital Institutional Review Board (IRB file no. 2016-11-034, 22.12.2016). VAN was administered at different doses according to body weight and symptoms, and the interval between the doses varied from 6 to 24 h. Blood samples were collected between 0.5 and 23.5 h after administration.

All information from the above studies, including the blood concentration of each drug and patient demographic data, was collected from hospital electronic medical records (EMRs), and patients were excluded if records of body weight, age and drug concentration were missing. The samples were collected when therapeutic drug monitoring was performed or when the drug concentration was expected to be at the trough level. In addition, drug concentrations were analyzed with a validated LC/MS/MS assay (API4000, Sciex, USA) for CsA or quality controlled turbidimetric immunoassays for PHB and VAN (PHB; Cobas 6000, Roche, Germany, VAN; TDx, Abott, USA) by department of diagnostics in the respective hospitals. 

### 2.3. Development of a Structural Model

Time-dependent blood concentrations were analyzed using the nonlinear mixed effect model in NONMEM 7.3 (ICON, USA) assisted by Perl-speaks NONMEM (PsN) 4.3.0 [34] and Xpose 4.0 [35]. Population PK parameters were estimated by first-order conditional estimation using the (FOCE + I) interaction method.

Structural PK parameters (*P_i_*) such as *k_a_*, *V_d_* and *CL* were included as fixed effects represented by $\theta_{i}$. Inter-individual variability (*IIV*) related to structural PK parameters were represented as exponentials *η_i_*. Estimated values of *IIV* was expressed as a coefficient of variation (CV%). For the residual variability (RV) represented by ε, the following models were evaluated and the most appropriate model was selected based on the objective function values: additive model, constant coefficient of variation (CCV) model and combined model. The relationships among structural PK parameters and *IIV* for each parameter could be described using Equation (3):

(3) $$P_{i}=\theta_{i}\times e^{\eta_{i}}.$$

To construct the structural model, one-, two- and three-compartment models were compared, and goodness of fit (GOF), objective function value (OFV) and visual predictive check (VPC) were calculated to assess model performance.

### 2.4. Incorporation of Size and Maturation Functions in the Structural Model

Size and maturation functions were included in the final structural model following stepwise analysis to reflect the growth and development of pediatric patients. To reflect the size, the allometric scaling method using normalized weight (Weight_normal_ = 70 kg) was applied to the structural PK parameters (Equation (4)), and the new parameter $P_{i}^{\prime }$ represented PK parameters in which size and maturity were considered. The sigmoidal *E_max_* function based on PCA or gestational age (GA) was applied to structural PK parameters to incorporate maturation using Equation (5):

(4) $$P_{i}^{\prime }=P_{i}\times {\left(\frac{\mathrm{Weight}}{{\mathrm{Weight}}_{\mathrm{normal}}}\right)}^{\mathrm{power}},$$

(5) $$P_{i}^{\prime }=P_{i}\times \frac{PCA^{Hill}}{PCA^{Hill}+TM_{50}{}^{Hill}}.$$

### 2.5. Steps for Covariate Searching

Covariate searching was performed using the stepwise covariate modeling (SCM) method after finalization of the structural model that included size and maturation functions [36]. To avoid covariate selection bias, covariates with >0.5 correlations with body weight and age were excluded from SCM steps [37]. Body surface area (BSA), glomerular filtration rate (GFR), serum creatinine (S_cr_), cystatin-C, blood urea nitrogen (BUN), aspartate transaminase (AST), alanine transaminase (ALT), serum albumin (ALB), total protein (TP), total bilirubin, direct bilirubin, total cholesterol and hematocrit (Hct) were evaluated as covariates. SCM was evaluated as forward selection with a *p*-value of 0.05 and backward selection with a *p*-value of 0.01. 

### 2.6. Model Evaluation

The model was tested to evaluate its bias, reliability of predictive power and model stability. For evaluating bias of the final model, goodness of fit (GOF) plot was used including individual prediction (IPRED) versus observation and conditional weighted residual (CWRES) versus time plot. VPC, one of the internal evaluation methods, was performed with 1000 samples, and 95% confidence intervals for the 5th, 50th and 95th percentiles of observation were obtained from the simulation results and visualized [38]. The non-parametric bootstrap evaluation method was performed 1000 times, and the 95% confidence interval for all parameters was obtained from the bootstrap results [39].

## 3. Results

### 3.1. Demographic Characteristics

Detailed demographics from all studies are summarized in Table 1. The mean postnatal age (PNA) of patients in CsA, PHB and VAN were 26.8 ± 17.8 months, 32.4 ± 30.7 days and 9.3 ± 12.4 weeks, respectively, and the body weight was 12.9 ± 3.8 kg, 3.3 ± 1 kg and 3.2 ± 2.6 kg, respectively. The compiled dataset from the three different clinical trials covered all ranges of the pediatric population and the preterm neonate to term neonate ratio was 79:21. 

### 3.2. Structural Model Development

**Figure 1:**
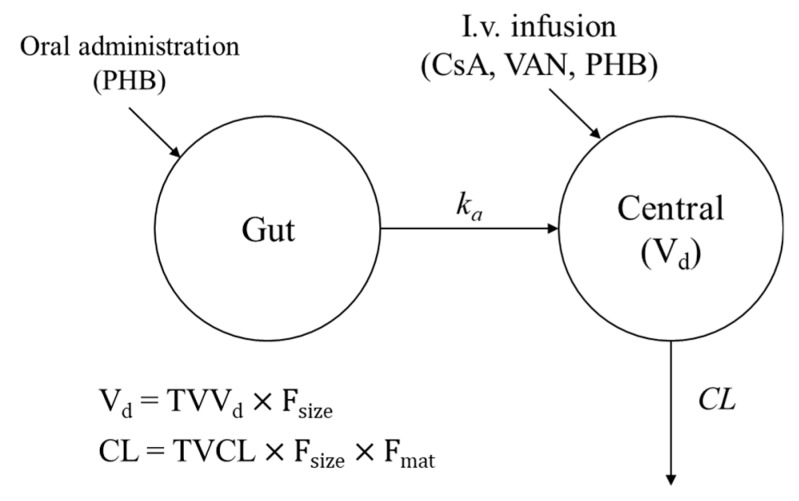
Pharmacokinetic model scheme for cyclosporine A (CsA), Vancomycin (VAN; Intravenous (i.v.) infusion) and phenobarbital (PHB; Oral administration and i.v. infusion).

The concentration of each drug was transformed to the natural logarithmic form before developing the model. One-, two- and three-compartment models were evaluated based on OFVs, and the one-compartment model was the most appropriate model for all three drugs. For population PK analysis of CsA and VAN, the one-compartment model with a first-order elimination was selected as the base model, whereas the one-compartment model with a first-order absorption (in the case of oral administration) and elimination was selected as the base model for population PK analysis of PHB (Figure 1). For the residual error model, the CCV model (Equation (6)) was selected as the final structural model which had numerical and graphical superiority compared to the other error models.

(6) $$C_{ij}=C_{pred,ij}\times \left(1+\varepsilon_{pro,ij}\right)$$

where *C_ij_* represents the observed *i*^th^ concentration in the *j*^th^ individual’s dose history and sampling time, and *ε_pro,ij_* (the residual error) are random variables with a mean of zero and variance *σ*^2^.

### 3.3. Covariate Searching for Size and Maturation Functions

The size function was applied to *CL* and *V_d_* (Equation (7)), and the maturation function was applied to *CL* (Equation (8)), to assess body growth and physical functional development in pediatric patients. However, in the case of CsA, the maturation function was not applied to *CL* because it was not statistically significant (Table 2)

(7) Vd=TVVd×(WTWTnormal)1×eη,

(8) CL={TVCL×(WTWTnormal)0.75×(AgeHillAgeHill+TM50Hill)×eη(PHB,VAN)TVCL×(WTWTnormal)0.75×eη(CsA).

The *TM*_50_ and the *Hill* coefficients, both parameters associated with the maturation function, were applied using reference values [2]. Other covariates not related to body size or maturation were tested, but they did not influence the PK parameters of V_d_ and CL.

### 3.4. Final Model Selection and Evaluation

**Figure 2:**
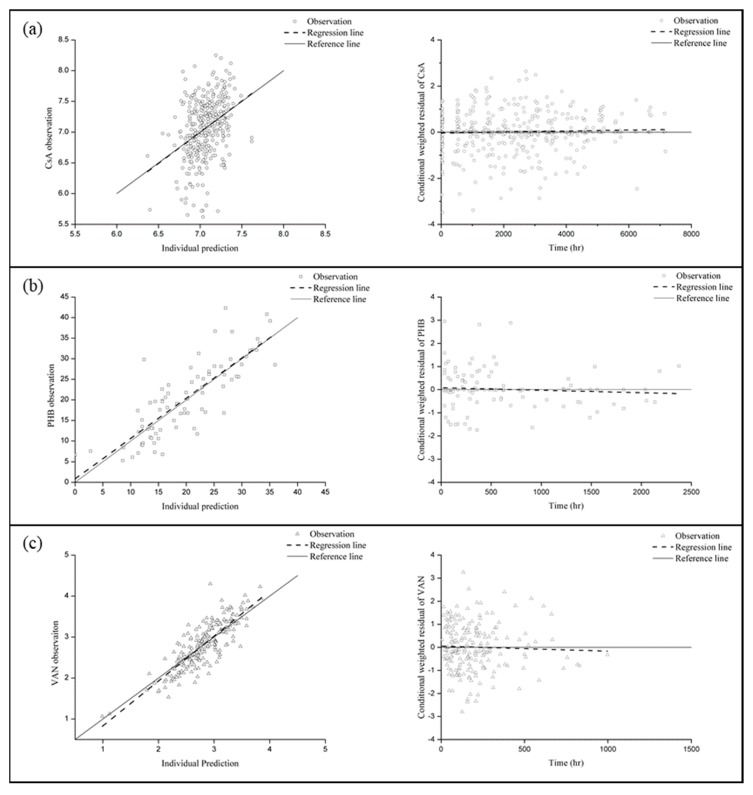
Goodness of fit plots of the final models. (**a**) Cyclosporine A (CsA), (**b**) phenobarbital (PHB), (**c**) vancomycin (VAN), open circle: CsA observation, open square: PHB observation, open triangle: VAN observation, solid line: reference line (y = x line for IPRED versus observation plot, y = 0 for CWRES versus time plot), dashed line: regression line.

Graphical and numerical criteria, such as OFVs and GOF plots, were compared to evaluate the ability of the models to explain the results for each drug. IPRED versus observation plots showed linearity and CWRES values of all models were in the acceptable range between −4 and 4 (Figure 2) [40].

**Figure 3:**
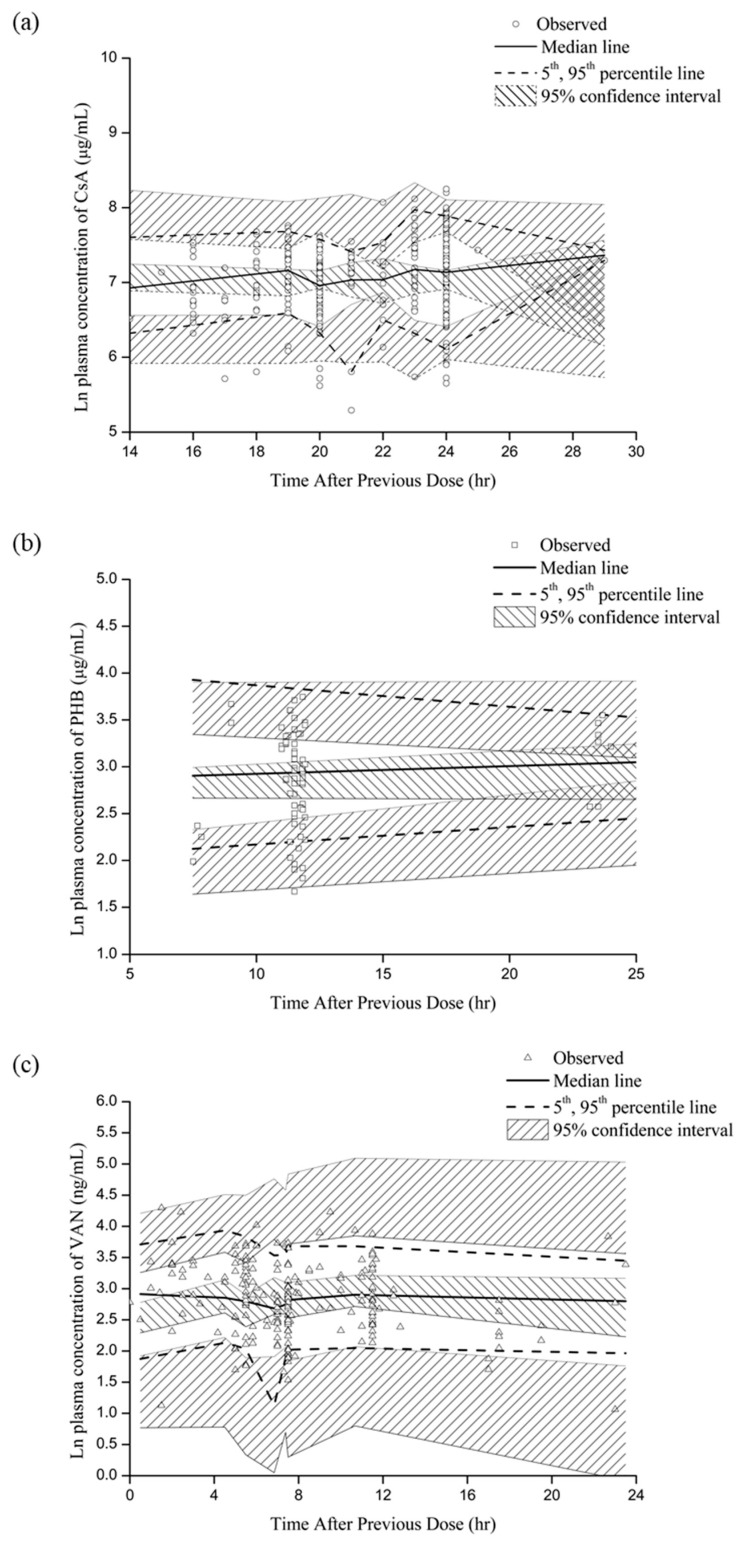
Visual predictive check for the final models of (**a**) cyclosporine A, (**b**) phenobarbital and (**c**) vancomycin. Open circles: observations, dashed lines: 5th and 95th percentiles, lines: median values, dashed area: 95% confidence intervals of 5th, 95th percentile and median values.

The estimated parameters of the final models and bootstrap results are summarized in Table 3. All PK parameters of the final model were within the 5th and 95th percentiles and were comparable with the median values based on bootstrap results. Furthermore, VPC showed that the observed data were within the predicted 95% confidence intervals for the 5th, 50th and 95th percentiles (Figure 3).

## 4. Discussion

In the present study, we performed population PK modeling to explore the PK characteristics following administration of CsA, PHB and VAN in pediatric patients. The model that incorporated both size scaling and maturation functions was found to provide an effective and efficient alternative method for optimizing drug dosage for pediatric patients. 

Overall, we recognized that *IIV* was higher than RV for the basic PK model without application of size and maturation functions. After application of these functions, the *IIV* dramatically decreased whereas only a slight decrease was observed with the RV. It could be an understandable phenomenon because size and maturation characteristics were more closely related to *IIV* than to RV. In addition, high RV is thought to originate from the high intra-individual differences which is an inherent characteristic of these drugs and the fact that the datasets were from samples that were collected sparsely from pediatric patients could also contribute to the high RV. Accordingly, the high RV values were deemed acceptable especially since the CRWES of all three models showed no bias which meant that selection of the error model was appropriate. 

For this study, CsA, PHB and VAN were selected for evaluation because they display high individual differences and narrow therapeutic ranges [41,42,43]. The CsA dataset mainly consisted of children patients (from infant to children), whereas the datasets for both PHB and VAN mainly included patients from preterm neonates to infants. Although all datasets were from pediatric patients and there were only small differences in the characteristics between them, the differences were significant enough to substantially affect the model development steps. Size scaling with allometric scaling methods, which is a factor representative of body size differences in pediatric patients, was applied to *V_d_* and *CL* parameters. In general, a change in body size can affect the distribution of drugs in the body, and this can be represented by *V_d_* [18,20]. Although *CL* is more closely related to maturation of organ functions than size, the size could affect the *CL* because many physiological factors such as liver metabolism display similar relationships between maturity and enzyme activity [19,21] and it was also impossible to statistically differentiate between the effects originating from maturation and size. For example, an increase in the number of enzymes can give similar results to an increase in enzyme ability due to maturation. On the other hand, the maturation function was only applied to *CL* and not to *V_d_* because there is no scientific evidence for a relationship between *V_d_* and maturation. 

The application of size function with allometric scaling to *CL* and *V_d_* was well implemented for all three drugs with statistical significance. Size with allometric scaling could account for body size differences in pediatric patients over one year of age (from children to adolescents). However, the estimation of allometric exponents for the PHB and VAN datasets including infants could explain the observed PK patterns better than when 0.75 was used as an exponent. This suggested that pediatric patients less than one year of age (from preterm neonates to infants) are affected by other factors such as maturity as well as growth and size. Thus, size scaling appeared to be applicable to pediatric patients of all ages, but the influence of size scaling was greater for patients greater than one year of age (from children to adolescents). 

The maturation function was applied to investigate the effect of size scaling on *CL*. When simultaneous usage of size scaling and maturation functions were applied to *CL* at the same time, PHB and VAN were statistically significant, but there was no such difference in the case of CsA. This may be an artifact due to the larger proportion of infants included within the PHB and VAN groups. In general, the activity of metabolic and excretory enzymes is increased until around one year of age during organ maturation, after which enzyme activity stabilizes [9,44]. Therefore, there are some limitations preventing a full explanation using size scaling alone when considering from preterm neonates to infants, and it appears to be better to simultaneously apply size scaling and maturation functions to *CL* when assessing the PK from preterm neonate to infants. As mentioned above, this could explain why the estimated exponents of *Power* were better than 0.75 in the case of preterm, term neonates and infants. 

## 5. Conclusions

Population PK models for pediatric patients were successfully developed which could thoroughly describe the PK of CsA, PHB and VAN following application of size and maturation functions. Both methods showed that size scaling is applicable to age groups of over one year of age (from children to adolescent), and the simultaneous usage of size and maturation functions is effective for predicting PK profiles in pediatric patients from preterm neonate to infant. In conclusion, the application of size scaling and maturation functions in pediatric population PK analysis can be highly effective and essential, but the application may be limited to certain age groups.

## Figures and Tables

**Table 1:** Demographic information for cyclosporine A (CsA), phenobarbital (PHB) and vancomycin (VAN).

Patient Characteristics	Number or Mean ± Standard Deviation (SD; Range)		
	CsA	PHB	VAN
No. of patients	34	28	93
Gender			
Male	20	11	57
Female	14	17	36
Age			
Gestational age	-	36.7 ± 4.4 (23.6–41.7) weeks	31.9 ± 4.7 (22.9–40.3) weeks
Postnatal age	26.8 ± 17.8 (1–79) months	32.4 ± 30.7 (3–150) days	9.3 ± 12.4 (0.1–80.4) weeks
Post-conceptional age	-	41.3 ± 3.9 (31–51.1) weeks	41.2 ± 14.2 (25.6–110) weeks
Body weight (kg)	12.9 ± 3.8 (5–24)	3.3 ± 1 (1–6.9)	3.2 ± 2.6 (0.4–14.9)
Birth weight (kg)	-	2.64 ± 0.87 (0.4–3.81)	-
Height (cm)	87.4 ± 14.4 (55–123)	50.6 ± 5.8 (31–63.2)	56.8 ± 5.4 (49.2–82.6)
Body surface area (m^2^)	-	-	0.2 ± 0.1 (0.1–0.5)
Serum creatinine (mg/dL)	0.34 ± 0.09 (0.2–0.8)	0.6 ± 0.59 (0.2–3.8)	0.4 ± 0.3 (0.1–3.37)
GFR (mL/min/1.73 m^2^)	142.8 ± 39.2 (63.4–250.4)	-	-
Cystatin-C (mg/L)	-	-	1.8 ± 0.5 (0.7–3.6)
AST (IU/L)	33.8 ± 9.0 (21–85)	64 ± 102.7 (11–676)	-
ALT (IU/L)	20.7 ± 112 (7–70)	65.7 ± 117.7 (7–765)	-
Blood urea nitrogen (mg/dL)	10.7 ± 3.8 (1.9–20.6)	-	-
Total bilirubin (mg/dL)	0.28 ± 0.33 (0.1–4.3)	3.8 ± 3.3 (0.2–14.5)	-
Direct bilirubin (mg/dL)	-	2.2 ± 2.7 (0.1–12.7)	-
Serum albumin (g/dL)	4.5 ± 0.3 (3.4–5.2)	-	2.7 ± 0.6 (1.6–4.9)
Total protein (g/dL)	-	-	4.4 ± 0.8 (1.7–6.9)
Haematocrit (%)	31.7 ± 3.4 (23.8–40.8)	-	-
Total cholesterol (mg/dL)	167.3 ± 29.7 (102–240)	-	-
Chemotherapy cycles (CTx)	5 ± 2.8 (1–12)	-	-

**Table 2:** Objective function value (OFV) of pharmacokinetic (PK) models for cyclosporine A (CsA), phenobarbital (PHB) and vancomycin (VAN).

Drug	Objective Function Value (ΔOFV)		
	Structural Model *	Structural Model + Size Scaling	Structural Model + Size Scaling + Maturation Function
CsA	−121.986 (-)	−153.115 (−31.129)	−155.075 (−33.089)
PHB	475.849 (-)	451.087 (−24.762)	400.966 (−74.883)
VAN	106.068 (-)	24.258 (−81.81)	−28.042 (−134.11)

* OFV standard.

**Table 3:** Estimated parameters from the final model and bootstrap results for cyclosporine A (CsA), phenobarbital (PHB) and vancomycin (VAN).

Parameters	CsA			PHB			VAN		
	Population Mean (%RSE)	IIV (CV%) (%RSE)	Bootstrap (*n* = 2000) 5th–95th Percentile	Population Mean (%RSE)	IIV (CV%) (%RSE)	Bootstrap (*n* = 2000) 5th–95th Percentile	Population Mean (%RSE)	IIV (CV%) (%RSE)	Bootstrap (*n* = 2000) 5th–95th Percentile
*CL* (L/hr)	21.3 (4.4%)	16.8% (17.5%)	19.8–22.9	0.569 (5.0%)	40.8% (1.2%)	0.34–4.82	69.4 (13.7%)	10.4% (68.2%)	49.5–89.2
*V_d_* (L)	218 (25.5%)	12.3% (110.7%)	91.6–344.8	5.51 (2.1%)	78.7% (6.8%)	1.87–13.53	3.23 (6.1%)	52.8% (15.9%)	2.9–3.6
TM_50_ (week)	-	-	-	48.2 (2.1%)	-	37.6–84.8	33.3 *	-	-
Hill coefficient	-	-	-	5.99 (1.2%)	-	1.6–8.3	3.68 *	-	-
*k_a_* (hr^−1^)	-	-	-	50 *	-	-	-	-	-
Bioavailability	-	-	-	0.724 (7.2%)	-	0.58–0.87	-	-	-
	**Proportional Error**								
**Residual variability**	46.8% (5.9%)	-	42.2–51.3%	35.6% (3.0%)	-	27.8–43.4%	40.8% (6.3%)	-	36.3–45.3%

IIV: Inter-individual variability; CV: Coefficient of Variance; RSE: Relative standard error; *: Fixed parameter.
